# Do Executive Function and Impulsivity Predict Adolescent Health Behaviour after Accounting for Intelligence? Findings from the ALSPAC Cohort

**DOI:** 10.1371/journal.pone.0160512

**Published:** 2016-08-01

**Authors:** Kaidy Stautz, Rachel Pechey, Dominique-Laurent Couturier, Ian J. Deary, Theresa M. Marteau

**Affiliations:** 1 Behaviour and Health Research Unit, University of Cambridge, Cambridge, United Kingdom; 2 Centre for Cognitive Ageing and Cognitive Epidemiology, University of Edinburgh, Edinburgh, United Kingdom; The University of Queensland, AUSTRALIA

## Abstract

**Objective:**

Executive function, impulsivity, and intelligence are correlated markers of cognitive resource that predict health-related behaviours. It is unknown whether executive function and impulsivity are unique predictors of these behaviours after accounting for intelligence.

**Methods:**

Data from 6069 participants from the Avon Longitudinal Study of Parents and Children were analysed to investigate whether components of executive function (selective attention, attentional control, working memory, and response inhibition) and impulsivity (parent-rated) measured between ages 8 and 10, predicted having ever drunk alcohol, having ever smoked, fruit and vegetable consumption, physical activity, and overweight at age 13, after accounting for intelligence at age 8 and childhood socioeconomic characteristics.

**Results:**

Higher intelligence predicted having drunk alcohol, not smoking, greater fruit and vegetable consumption, and not being overweight. After accounting for intelligence, impulsivity predicted alcohol use (odds ratio = 1.10; 99% confidence interval = 1.02, 1.19) and smoking (1.22; 1.11, 1.34). Working memory predicted not being overweight (0.90; 0.81, 0.99).

**Conclusions:**

After accounting for intelligence, executive function predicts overweight status but not health-related behaviours in early adolescence, whilst impulsivity predicts the onset of alcohol and cigarette use, all with small effects. This suggests overlap between executive function and intelligence as predictors of health behaviour in this cohort, with trait impulsivity accounting for additional variance.

## Introduction

Individual differences in cognitive resource, an umbrella term referring to general cognitive ability (intelligence) as well as more specific abilities such as executive functioning and impulse control, are a potential contributor to health-related behaviours and health outcomes. A better understanding of how cognitive resource influences health behaviour may help to design effective interventions to improve population health. Despite an abundance of research examining links between distinct aspects of cognitive resource and health behaviours, there have been limited studies with representative samples that include multiple aspects of cognitive resource to account for their shared variance. It is therefore not known which aspects of cognitive resource are most relevant for understanding health behaviours. This study investigates whether executive function and impulsivity, assessed in childhood, are unique predictors of health-related behaviours and overweight in adolescence after accounting for intelligence.

The term executive function refers to a set of cognitive processes involved in planning, monitoring, and revising goal-directed behaviour. An influential framework of executive function specifies three such processes: response inhibition, updating working memory, and shifting between mental sets [1]. Abilities such as attentional control and planning are also regarded as aspects of executive function in some taxonomies, though these higher order abilities are thought to be influenced by the three ‘pure’ executive processes [2, 3].

When assessed in older adulthood, stronger executive function predicts longer life expectancy and a lower occurrence of chronic illness [4, 5]. One mechanism that may underlie these relationships is an association between executive function and health-related behaviours, a mediator of life expectancy [6]. Evidence is accumulating that executive function is positively associated with healthy behaviours such as eating fruit and vegetables and partaking in physical activity, and negatively associated with unhealthy behaviours such as drinking alcohol, smoking, and eating high-fat foods [7–10]. These associations are observable in childhood and adolescence. Amongst children, components of executive function predict fruit and vegetable intake and physical activity, are negatively correlated with high-calorie snack consumption and sedentary behaviour, and are weaker in obese individuals compared to those of healthy weight [11, 12]. Adolescents with weak response inhibition and working memory are more likely to initiate alcohol use earlier, drink alcohol more frequently, and be obese [13–15].

Weak executive function is reflected to some degree in the construct of impulsivity, a tendency to show poor planning, persistence, attention, and inhibition [16]. Though distinct, executive function and impulsivity show overlap in how they are described and operationalised. Executive function and ‘state’ impulsivity are typically assessed as specific mental capabilities using an overlapping set of behavioural tasks [17, 18]. However, executive function includes components not related to impulsivity, such as working memory, and impulsivity is also studied as a personality trait using self- or other-rated scales which assess many behavioural tendencies, some of which are unrelated to executive function [17, 19]. Studies examining trait impulsivity as a predictor of health behaviours show similar results to those focusing on executive function: individuals higher in impulsivity are more likely to drink alcohol, smoke, and eat unhealthily, with small to medium effect sizes [19, 20].

Executive function also shows conceptual overlap with intelligence. Diamond [2] suggests that fluid intelligence reflects a higher order executive function, responsible for complex cognition such as reasoning and problem-solving. Intelligence consistently shows a large association with working memory, though smaller associations with other aspects of executive function [21–23]. Neuropsychological evidence suggests that intelligence and executive function depend upon shared, though not identical, neural substrates [24]. Intelligence has also been shown to be negatively associated with trait impulsivity [25, 26].

Evidence from longitudinal cohort studies indicates that higher intelligence in childhood and adolescence is associated with decreased mortality risk [27], as well as engagement in healthy behaviours such as fruit and vegetable consumption, physical activity, not smoking, and not being obese in adulthood [28–30]. Conversely, higher childhood intelligence predicts a *greater* likelihood of alcohol use in adolescence [31]. Given the conceptual and psychometric overlap between intelligence and executive function, and the similar patterns of association between these constructs and certain health-related behaviours, it is possible that the utility of executive function as a predictor of health-related behaviour can be explained by intelligence.

Three previous studies have found evidence that executive function prospectively predicts unhealthy behaviours after accounting for intelligence. Aytaclar et al. [32] found that low executive function (a latent factor reflecting scores on six tasks) at age 10 predicted smoking at age 12 independently of verbal intelligence (assessed with the Wechsler Intelligence Scale for Children [WISC]) in 275 participants, 106 of whom had a parent with a substance use disorder. Executive function did not predict alcohol use in this study. Nigg et al. [33] found that response inhibition predicted alcohol-related problems independently of intelligence (assessed with the revised WISC) in 498 adolescents, around half of whom had a parent with an alcohol use disorder. Deckel and Hesselbrock [34] found that low scores on an executive function factor reflecting scores on three tasks predicted increases in alcohol use independently of intelligence (assessed with the Wechsler Adult Intelligence Scale), but only in participants with a positive family history of alcoholism in a sample of 104 adults, 66 of whom had a parent with an alcohol use disorder. These studies are informative, but their use of relatively small, non-representative samples limits the degree to which these findings might generalise to other populations. Also, the tendency to group executive function scores into composite factors may obscure the specific executive processes driving health-related behaviours.

Associations between impulsivity and health behaviours may also be partially explained by intelligence. Few studies to our knowledge have examined both impulsivity and intelligence as prospective predictors of health behaviours. Moffitt et al. [35] used a large sample of 1037 participants followed up for over 22 years, and found that trait impulsivity in childhood predicted early smoking and adult smoking dependence, but not alcohol dependence, independently of intelligence. This study did not assess components of executive function. In the present study, we aim to build upon previous findings by using data from a large, representative cohort that includes measures of multiple components of executive function and impulsivity, as well as intelligence. Furthermore, we will assess associations with healthy behaviours–fruit and vegetable consumption and physical activity–as well as unhealthy behaviours.

The aim of this study is to assess whether four measured components of executive function (selective attention, attentional control, working memory, and response inhibition) and the personality trait of impulsivity, assessed between ages 8 and 10, predict four health-related behaviours (alcohol use, smoking, eating fruit and vegetables, and physical activity) and overweight at age 13, after accounting for intelligence measured at age 8 and childhood socioeconomic status. We predict that high executive function and low impulsivity will predict engaging in healthy behaviours, not engaging in unhealthy behaviours, and not being overweight in adolescence, even after accounting for intelligence.

## Method

### Participants

Data come from the Avon Longitudinal Study of Parents and Children (ALSPAC), a population-based cohort of pregnant women with due dates between 1^st^ April 1991 and 31^st^ December 1992 who were resident in the Avon Health Authority (South West England) at the time of recruitment. Enrolled women had 14,062 live-born children, who form the core ALSPAC sample.

The measures used in the current study were taken during clinical assessments to which all children with known current addresses were invited. The number of eligible participants (excluding those that had died, withdrawn from the study, or were untraceable) invited to these assessments was 14,057 at ages 7 to 12, and 12,782 at age 13 [36]. The number of attendees at each of the clinics was as follows (name given to that ALSPAC clinic in parentheses): 7488 at age 8 (Focus@8); 7563 at age 10 (Focus@10); and 6147 at age 13 (TF2).

Participants with complete data for any of the five outcomes of interest at age 13 were included. Table 1 presents the number of participants with complete data for each outcome. For pregnancies involving more than one child only one child was randomly selected for analysis to achieve data independence.

**Table 1:** Number of participants with complete data.

	Alcohol use	Smoking	Fruit and vegetable consumption	Physical activity	BMI
**Participants with complete outcome data**	6033	6034	6024	4273	6042
**Participants with complete data on all variables (%)**	3106 (51.2)	3106 (51.2)	3099 (51.1)	2254 (37.1)	3110 (51.2)

### Measures

Table 2 presents sample characteristics and mean scores for measures of intelligence, impulsivity, and executive function.

**Table 2:** Participant characteristics and scores on cognitive variables.

Variable	Child’s age at assessment	N (%)	Mean (SD)
**Gender**	Birth		
Male		2981 (49.1%)	
Female		3088 (50.9%)	
**Ethnicity**	Birth		
White		5295 (87.2%)	
Non-White		213 (3.5%)	
Missing		561 (9.2%)	
**Mother’s highest educational qualification**	32 weeks		
CSE		688 (11.3%)	
Vocational		457 (7.5%)	
O Levels		1974 (32.5%)	
A Levels		1526 (25.1%)	
Degree		951 (15.7%)	
Missing		473 (7.8%)	
**Household income (£/week)**	33 months		
0–100		266 (4.4%)	
101–200		711 (11.7%)	
201–300		1361 (22.4%)	
301–400		1116 (18.4%)	
401+		1272 (21.0%)	
Missing		1343 (22.1%)	
**Intelligence (WISC-III–full scale IQ score)**	8 years	5222	105.69 (16.26)
**Impulsivity (Parent-rated Strengths & Difficulties Questionnaire– 0–10 scale)**	9 years	5182	2.85 (2.22)
**Executive function:**			
**Selective attention (Sky Search task–delay in seconds)**	8 years	5089	5.16 (1.85)
**Attentional control (Opposite Worlds task–delay in seconds)**	8 years	5014	0.55 (0.20)
**Working memory (Counting Span task–correctly recalled sets)**	10 years	5186	3.42 (0.85)
**Response inhibition (Stop Signal task–stop signal reaction time)**	10 years	5103	250.47 (91.17)

#### Intelligence

The Wechsler Intelligence Scale for Children, Third Edition (WISC-III) was administered to participants at age 8. A short form of the scale, whereby only alternate items are completed, was used. The scale comprises five verbal subtests and five performance subtests. The full scale total Intelligence Quotient (IQ) score was used in our analysis. The WISC-III has been shown to be a highly reliable and valid measure of intelligence [37].

#### Impulsivity

The hyperactivity/impulsivity component of the Strengths and Difficulties Questionnaire [38] was completed by the child’s parent when children were aged 9 (115 months). This scale comprises five items, each with three response options (not true/somewhat true/certainly true), with total scores ranging from 0 (low) to 10 (high impulsivity). Example items are *“Thinks things out before acting”* and *“Sees tasks through to the end*, *good attention span”*. The reliability and validity of the Strengths and Difficulties Questionnaire is well-established [39]. We note that this assessment of impulsivity was a rated personality trait and not a measured cognitive function.

#### Executive function

**Selective attention:** The Sky Search task from the Test of Everyday Attention for Children (TEA-Ch [40]) was completed at age 8. This task examines the ability to attend to a task whilst rejecting irrelevant or distracting information. The task involved two trials. In the first trial, children were asked to circle pairs of identical spaceships from an array of identical and non-identical spaceships as quickly as possible. In the second trial, included to adjust for differences in motor ability, participants repeated the procedure with a new array containing only identical pairs of spaceships. The final score is the average time taken to find each pair of spaceships in the first trial (calculated as time taken in seconds divided by the number of spaceship pairs correctly circled) minus average time to find each pair in the second trial. Scores were reflected for analysis so that higher scores reflect better performance. There were no data to calculate internal consistency.

**Attentional control:** The Opposite Worlds task from the TEA-Ch was completed at age 8. In this task participants are required to give verbal responses that contradict the visual information they are presented. Children were shown a path made up of the numbers 1 and 2, with 24 numbers in total. In the ‘same world’ (control) condition, children had to call the numbers out as quickly as possible while the examiner kept their finger next to each number in the path until the child read it correctly. In the ‘opposite world’ condition, the children had to inhibit the familiar response and call out ‘two’ when they reach a 1 and ‘one’ when they reach a 2. Children were given a demonstration of each condition and completed a practice attempt at each before being reminded of the rules. There were four test trials presented as follows: same, opposite, opposite, same. The final score was the mean time taken for the opposite worlds condition minus the mean time taken for the same worlds condition, reflecting the degree to which performance was impaired in the opposite worlds condition.

**Working memory:** The Counting Span Task was completed at age 10. The task was presented on a computer. Children were shown a number of red and blue dots on a white screen, and asked to point to and count the number of red dots out loud. Children completed two practice sets of two screens, followed by three sets each of two, three, four, and five screens. After each set, children were asked to recall the number of red dots seen on each screen, in the order they were presented within that set. All children worked through all of the sets. Working memory span was calculated as the number of correctly recalled sets weighted by the number of screens within each set. Previous studies indicate that this task has good internal consistency and acceptable test-retest reliability [41].

**Response inhibition:** Children completed the Stop Signal Task [42] at age 10. Children were asked to sit in front of a computer monitor and their two index fingers were placed in two stimulus boxes, one labeled X and one labeled O. Two types of trials were performed, primary trials and stop signal trials. In the primary trials children were asked to fixate on a small smiley face presented in the centre of the computer screen. An X or O was presented on the screen and the child had to press the corresponding button as quickly as possible. Thirty trials were completed (15 X and 15 O). Mean response times were calculated. The stop signal trials were as above but with an audible beep (stop signal) occurring after presentation of the X or O on certain trials. The child was told to not press a response button when the beep was sounded, inhibiting the learned response. The beep sounded on random trials at 150 or 250ms before the child’s mean reaction time to the primary trials. Participants completed 24 practice trials, followed by two experimental blocks. These blocks consisted of 48 trials in each, 16 of which contained stop signals. An estimate of stop signal reaction (SSRT) was calculated (see [42]). SSRT for the more difficult condition (150ms delay) was used as our response inhibition measure, due to a ceiling effect for the 250ms delay condition. Scores were reversed so that higher scores reflect better inhibition ability.

Set shifting ability was not assessed in the ALSPAC cohort and was therefore not analysed in the current study.

#### Health behaviours at age 13

**Alcohol use:** Participants were asked if they had ever drunk a whole alcoholic drink, with a dichotomous (yes/no) response format.

**Smoking:** Participants were asked if they had ever smoked a cigarette in their lifetime, with a dichotomous (yes/no) response format.

**Fruit and vegetable consumption:** Children were asked to record, with the help of their carer, a diary of all food and drink consumed over two weekdays and one weekend day. Days were self-selected and not necessarily consecutive. A full description of food and drink consumed, using household measures, and description of any leftovers was requested. Children were asked to bring their completed diaries to the clinic, where they were interviewed by a nutrition fieldworker to gain further information including portion size and cooking methods. If no diary was brought to the clinic, the child was asked about everything consumed during the previous 24 hours. The measure used for this study was the average amount of fruit and vegetables, in grams, consumed per day over the three day period.

**Physical activity:** Average daily minutes of moderate/vigorous physical activity, objectively recorded using Actigraph monitors which children wore for one week during waking hours. A minimum of wearing the monitor for 10 hours on at least 3 days was required for data inclusion.

**Body Mass Index (BMI):** Derived from height and weight, measured by a nurse. The mean BMI was 20.38 (SD = 3.52). A continuous score was used for correlation analysis. For the prediction model we wanted to investigate predictors of overweight status. As overweight status by BMI depends on age and gender in children and adolescents, we created a dichotomous variable (either not overweight or overweight/obese) based on recommended age- and gender-specific thresholds [43].

#### Covariates

Three demographic measures were included: *age*, *gender*, and *ethnicity* (coded dichotomously as white/non-white). Ethnicity was included as a marker of socioeconomic position that has been shown to impact upon both cognitive resource and health [44, 45]. There is evidence for stronger associations between impulsivity and alcohol use, for instance, among young people from white, compared to non-white ethnic backgrounds [46, 47]. Two additional markers of childhood socioeconomic status were included. These were treated as ordinal variables and dummy coded for analysis. *Maternal education* was mother’s highest educational qualification (CSE, Vocational, O Level, A Level, or Degree) at 32 weeks gestation. *Household income* was the average take-home family income per week, including benefits, assessed when the child was 33 months old. Scores were divided into quintiles, with *5* being the highest.

### Data analysis

#### Missing data

A total of 6069 participants had complete data on at least one outcome measure. These participants formed our analytic sample. Missing data for these participants were imputed using Multivariate Imputation by Chained Equations (MICE). This method involves creating multiple 'complete' datasets by imputing missing values multiple times, based on (a) observed values for a given individual, and (b) the associations between variables in the imputation model observed for participants with complete data. A detailed overview of the MICE procedure has been reported previously [48, 49]. One set of 75 imputations was performed. Data were assumed to be missing at random (MAR), meaning that the probability of a value being missing only depends on variables included in the imputation model [50]. Based on previous findings from this cohort [51] variables related to socioeconomic status were included in the imputation process to make the MAR assumption more tenable. Our imputation models therefore included all of the variables included in our prediction models (see ‘Regression analysis‘ below).

#### Correlational analysis

To assess bivariate associations Spearman correlations were calculated for continuous variables, tetrachoric correlations for dichotomous variables, and biserial correlations for associations between continuous and dichotomous variables. Spearman correlations were used rather than Pearson correlations due to multiple variables showing non-normal distributions. Correlational analysis was conducted on data from complete cases only and on the average values of the 75 imputed datasets. To assess whether observed correlations were significantly different from zero, non-parametric bootstrapping with 10,000 replications was used. Bootstrapping is a method for estimating robust standard errors and confidence intervals of a parameter in complex situations where parametric inference is not possible (see [52] for an introduction and overview to bootstrapping methods).

#### Regression analysis

Alcohol use, smoking, and overweight were dichotomous (yes/no) variables and were analysed using logistic regression models. Fruit and vegetable consumption scores were semi-continuous with a large number of zero values, and were analysed using a zero-adjusted gamma regression model. These are three-component models that separately predict the probability of scoring zero (modelled using logit link), mean scores amongst those scoring over zero (zeros-excluded log link), and the variance amongst those scoring over zero. We present results pertaining to the first two components only, as these specifically address our research questions. Physical activity scores were semi-continuous and were initially analysed using a zero-adjusted gamma regression model. As only a very small number of participants scored zero (*n* = 10), we decided that the logit link component of this model was not informative. We therefore excluded these participants and employed a gamma regression model to predict non-zero physical activity scores (*n* = 6059). As a sensitivity analysis we included these participants in the gamma regression model with their scores adjusted to low values higher than zero. The pattern of results was unchanged.

Three models were constructed for each outcome and analysed separately. Model 1 tested the effect of intelligence, not adjusting for executive function or impulsivity. Model 2 tested the effects of executive function and impulsivity, not adjusting for intelligence. In Model 3, intelligence, impulsivity and executive function were entered simultaneously. Covariates were included in all three models. Wald tests were conducted to assess whether Model 3 showed statistically improved fit over Models 1 and 2.

As intelligence, executive function, and health-related behaviours have all been shown to be associated with socioeconomic position [53, 54], models were tested with and without socioeconomic indicators to assess whether effects were attenuated with these variables included.

The Bonferroni correction for multiple comparisons was employed. As five outcomes were analysed, the alpha level was set to .01. We report 99% confidence intervals to reflect this adjusted alpha level.

## Results

### Sample characteristics

Table 3 reports the percentage of the analytic sample that reported ever drinking an alcoholic drink, ever smoking, or being overweight or obese, as well as mean scores for fruit and vegetable consumption and moderate to vigorous physical activity.

**Table 3:** Sample characteristics for health-related behaviours.

	Percentage	Mean (Standard deviation)
Ever drunk an alcoholic drink	52.6	
Ever smoked a cigarette	19.2	
Grams of fruit and vegetables consumed per day		340.7 (260.1)
Moderate-vigorous physical activity per day		24.0 (17.3)
Overweight or obese	19.3	

### Correlations

Table 4 presents bivariate correlations between cognitive variables (intelligence, executive function, and impulsivity) and health-related behaviours for complete cases. Comparison of these coefficients with the mean values of imputed datasets indicated a high degree of consistency, with 0.015 being the maximum absolute difference between non-imputed and imputed coefficients. In line with previous studies [23] we found that working memory was the aspect of executive function with the largest association with intelligence (*rho* = .38). Response inhibition showed very small correlations with all other variables. Cognitive variables showed small associations with health-related behaviours on the whole, with all correlations lower than .20 in absolute values. Wechsler intelligence and parent-rated impulsivity correlated at *rho* = -.22.

**Table 4:** Pairwise correlations between cognitive variables and health-related behaviours.

	1	2	3	4	5	6	7	8	9	10	11
**1. Intelligence**	-										
**2. Impulsivity**	-.22***	-									
**Executive function:**											
**3. Selective attention**	.21***	-.11***	-								
**4. Attentional control**	.23***	-.09***	.21***	-							
**5. Working memory**	.38***	-.17***	.12***	.20***	-						
**6. Response inhibition**	-.03*	-.06***	-.05**	-.01	.01	-					
**Health-related behaviours:**											
**7. Alcohol use**	.05**	.03	.03	.04*	.04*	-.03	-				
**8. Smoking**	-.11***	.12***	.00	-.01	-.09***	-.01	.58***	-			
**9. Fruit and vegetable consumption**	.18***	-.09***	.02	.02	.11***	.00	-.02	-.08***	-		
**10. Physical activity**	.00	.07***	-.07***	.00	.00	.01	.08***	.09***	.03*	-	
**11. Body mass index**	-.05***	.01	.03*	-.03	-.06***	-.01	.08***	.08***	-.03**	-.12***	-

* *p* < .05

**p < .01

***p < .001

Calculated *p* values are for two-sided tests.

### Prediction of health-related behaviours and overweight

Table 5 displays the *p* values of Wald tests comparing Model 3 (full model with intelligence, executive function, and impulsivity included) with both Model 1 (intelligence only) and Model 2 (executive function and impulsivity only). The full models, containing all cognitive variables, showed improved fit over models containing only intelligence for all outcome variables except physical activity.

**Table 5:** Significance level (*p* values) of model comparison Wald tests.

	Model 3 (full model) compared to Model 1 (intelligence only)	Model 3 (full model) compared to Model 2 (intelligence not included)
Alcohol use	**.0027**	**.0009**
Smoking	**< .0001**	.1168
Overweight	**.0004**	**.0003**
Fruit and vegetable consumption	**.0047**	**< .0001**
Physical activity	.2294	.7209

Results of Wald tests comparing Model 3 (full model including intelligence, executive function, and impulsivity) with Model 1 (intelligence only) and Model 2 (executive function and impulsivity only) for each outcome variable. Bold values indicate that Model 3 was a significant improvement.

Table 6 presents the results of models predicting alcohol use, smoking, and overweight. Table 7 presents the results of models predicting fruit and vegetable consumption and physical activity. Comparison of models that included or excluded socioeconomic variables indicated modest attenuation of intelligence effects in models that included these variables, yet a highly similar pattern of results overall. We present results from models that included these variables.

**Table 6:** Logistic regressions predicting alcohol use, smoking, and overweight at age 13.

	Alcohol use			Smoking			Overweight		
	Odds Ratio	99% CI	*p*	Odds Ratio	99% CI	*p*	Odds Ratio	99% CI	*p*
**Model 1**									
**Intelligence**	**1.13**	**1.05, 1.23**	**< .001**	**0.88**	**0.79, 0.97**	**.001**	**0.88**	**0.79, 0.97**	**< .001**
**Model 2**									
**Impulsivity**	**1.08**	**1.01, 1.17**	**.005**	**1.23**	**1.12, 1.35**	**< .001**	1.03	0.94, 1.13	.363
**Executive function:**									
***Selective attention***	1.04	0.96, 1.12	.192	1.01	0.91, 1.11	.892	1.05	0.95, 1.16	.216
***Attentional control***	1.06	0.98, 1.14	.06	1.02	0.93, 1.12	.536	**0.90**	**0.81, 0.99**	**.006**
***Working memory***	**1.08**	**1.004, 1.17**	**.007**	0.92	0.83, 1.01	.019	**0.87**	**0.79, 0.97**	**< .001**
***Response inhibition***	0.96	0.89, 1.03	.134	0.98	0.89, 1.07	.514	0.92	0.84, 1.01	.025
**Model 3**									
**Intelligence**	**1.12**	**1.03, 1.23**	**.001**	0.93	0.83, 1.05	.117	0.92	0.82, 1.02	.042
**Impulsivity**	**1.10**	**1.02, 1.19**	**.001**	**1.22**	**1.11, 1.34**	**< .001**	1.02	0.93, 1.12	.604
**Executive function:**									
***Selective attention***	1.02	0.94, 1.10	.529	1.02	0.92, 1.12	.660	1.07	0.96, 1.18	.112
***Attentional control***	1.04	0.97, 1.13	.142	1.03	0.94, 1.13	.421	0.90	0.82, 1.004	.013
***Working memory***	1.05	0.97, 1.14	.113	0.93	0.84, 1.03	.077	**0.90**	**0.81, 0.99**	**.006**
***Response inhibition***	0.96	0.90, 1.03	.167	0.98	0.89, 1.07	.482	0.92	0.84, 1.01	.020

*N* = 6069. Models adjusted for demographic and socioeconomic variables (age, gender, ethnicity, maternal education, household income). Bold values indicate *p* < .01. Odds ratios indicate the difference in outcome for a 1 standard deviation increase in predictor variables.

**Table 7:** Gamma regressions predicting fruit and vegetable consumption and moderate-vigorous physical activity at age 13.

	Fruit and vegetable consumption						Physical activity		
	Odds of no consumption (logit link)			Mean (zeros excluded, log link)			Mean (log link)		
	Odds Ratio	99% CI	*P*	*β*	99% CI	*p*	*β*	99% CI	*p*
**Model 1**									
**Intelligence**	**0.71**	**0.59, 0.86**	**< .001**	**.056**	**.028, .084**	**< .001**	-.002	-.033, .029	.865
**Model 2**									
**Impulsivity**	1.14	0.97, 1.34	.043	**-.032**	**-.058, -.006**	**.003**	.016	-.015, .047	.161
**Executive function:**									
***Selective attention***	1.06	0.89, 1.27	.380	-.008	-.036, .002	.454	.002	-.029, .033	.879
***Attentional control***	1.07	0.90, 1.27	.303	-.016	-.044, .012	.141	-.017	-.048, .014	.158
***Working memory***	0.84	0.70, 1.01	.015	**.033**	**.007, .059**	**.001**	.024	-.007, .055	.047
***Response inhibition***	0.96	0.81, 1.03	.533	.008	-.018, .034	.441	.008	-.023, .039	.549
**Model 3**									
**Intelligence**	**0.71**	**0.57, 0.87**	**< .001**	**.052**	**.021, .083**	**< .001**	-.005	-.041, .031	.721
**Impulsivity**	1.08	0.91, 1.28	.244	-.024	-.052, .004	.025	.015	-.016, .046	.184
**Executive function:**									
***Selective attention***	1.13	0.94, 1.37	.090	-.017	-.045, .011	.126	.003	-.030, .037	.831
***Attentional control***	1.11	0.94, 1.31	.124	-.021	-.049, .007	.051	-.016	-.047, .015	.171
***Working memory***	0.92	0.76, 1.12	.293	.020	-.008, .048	.062	.025	-.009, .059	.047
***Response inhibition***	0.95	0.81, 1.12	.438	.009	-.017, .035	.357	.007	-.024, .038	.555

*N* = 6069. Models adjusted for demographic and socioeconomic variables (age, gender, ethnicity, maternal education, household income). Bold values indicate *p* < .01. Odds ratios and standardised beta coefficients indicate the difference in outcome for a 1 standard deviation increase in predictor variables.

#### Alcohol use

In Model 1, higher intelligence predicted a greater likelihood of ever having drunk alcohol. In Model 2, higher impulsivity and higher working memory predicted a greater likelihood of having drunk alcohol. In Model 3, which showed improved fit over both Models 1 and 2, intelligence and impulsivity remained significant predictors of having drunk alcohol but working memory did not.

#### Smoking

In Model 1, higher intelligence predicted a lower likelihood of ever having smoked. Children whose mothers attained a degree showed a reduced likelihood of smoking compared to those whose mothers attained a CSE qualification (Odds Ratio [OR] = 0.59, 99% Confidence Interval [CI] = 0.04, 0.85, *p <* .001). Compared to those in the 1^st^ (lowest) quintile, children with household income in the 3^rd^ (OR = 0.67, 99% CI = 0.46, 0.99, *p* = .009) and 4^th^ quintiles (OR = 0.65, 99% CI = 0.43, 0.97, *p* = .005) were less likely to smoke. In Model 2, higher impulsivity predicted a greater likelihood of smoking. In Model 3, which showed improved fit over Model 1 but not Model 2, impulsivity remained a significant predictor of smoking but intelligence did not.

#### Overweight

In Model 1, higher intelligence predicted a lower likelihood of being overweight. Children whose mothers attained a degree showed a reduced likelihood of being overweight compared to those whose mothers attained a CSE qualification (OR = 0.53, 99% CI = 0.37, 0.77, *p* < .001). In Model 2, higher working memory and attentional control predicted a lower likelihood of being overweight. In Model 3, which showed improved fit over Models 1 and 2, working memory remained a significant predictor of overweight but intelligence and attentional control did not.

#### Fruit and vegetable consumption

In Model 1, higher intelligence predicted a greater likelihood of eating any fruit and vegetables compared to none, and predicted higher mean consumption amongst participants who ate any. Compared to children whose mothers attained a CSE qualification, those whose mothers attained O Levels (Standardised beta [*β*] = .170, 99% CI = .077, .263, *p* < .001), A Levels (*β* = .289, 99% CI = .194, .384, *p* < .001), or degrees (*β* = .367, 99% CI = .261, .473, *p* < .001) consumed progressively greater amounts of fruit and vegetables. Compared to those in the 1^st^ (lowest) quintile, children with household income in the 5^th^ (highest) quintile consumed greater amounts of fruit and vegetables (*β* = .157, 99% CI = .020, .294, *p* = .003). In Model 2, higher working memory predicted higher mean fruit and vegetable consumption. In Model 3, which showed improved fit over Models 1 and 2, intelligence remained a significant predictor but working memory did not.

#### Physical activity

Across all models there were no significant predictors of mean moderate to vigorous physical activity.

### Sensitivity analysis

Analyses were conducted on participants with complete data on all outcomes and covariates only (*N* = 2,554–3,110 depending on outcome), and again on a dataset with imputed missing values for all ALSPAC participants (*N* = 14,057). Similar estimates were obtained for each analysis, with the only notable difference being smaller standard errors in the fully imputed dataset.

## Discussion

This study investigated whether executive function and impulsivity assessed in childhood predict health-related behaviours and overweight at age 13 after accounting for intelligence. Higher intelligence predicted alcohol use, not smoking, not being overweight, and greater fruit and vegetable consumption. Executive function test scores did not predict health-related behaviours after accounting for intelligence but did predict overweight status, with stronger working memory predicting not being overweight. The parent-rated personality trait of impulsivity predicted initiation of alcohol use and smoking. The size of these effects was small.

In line with previous evidence, childhood intelligence predicted multiple health-related behaviours. It is noteworthy that individuals with higher intelligence had higher odds of drinking alcohol by age 13, consistent with research showing that high intelligence predicts more frequent alcohol use and greater quantities consumed in adolescents [31]. Evidence of links between childhood intelligence and alcohol use later in life is mixed, however, with some epidemiological studies showing that more intelligent children go on to have a heavier intake of alcohol and a higher likelihood of experiencing alcohol-related problems in adulthood [55], and others showing that more intelligent children go on to drink less in adulthood [56]. It has been suggested that higher verbal intelligence may lead to earlier alcohol use initiation via an increased tendency to associate with alcohol-using peers [57]. Alcohol use by more intelligent adolescents may therefore start out as a useful strategy for making friends and elevating social status, but with the potential to set longer term drinking habits and increase intake across the lifespan.

Our hypothesis that components of executive function would predict adolescent health-related behaviours after accounting for intelligence was not supported, although stronger childhood working memory did predict a lower likelihood of being overweight at age 13.

This finding adds to a small literature that has identified an association between working memory ability and weight status in children and adolescents [58]. Children with low working memory have been found to be more likely to eat high fat foods [59]. However, the direction of influence is as yet unclear as few previous investigations have found longitudinal evidence of poor working memory predicting later overweight/obesity, as the current study did. There may be a reciprocal influence, with high fat diets causing detriments to working memory which in turn make unhealthy eating habits more likely.

Before accounting for intelligence, stronger working memory predicted alcohol use and greater fruit and vegetable consumption, and stronger attentional control predicted a lower likelihood of being overweight. These associations were no longer present after accounting for intelligence. It is possible that previously identified links between components of executive function and health-related behaviours in this age group can be explained by the variance shared between executive function and intelligence. Regarding alcohol use specifically, previous studies that have observed executive function to predict later alcohol use outcomes after accounting for intelligence only found effects amongst children already at increased risk due to familial alcohol use disorders [34]. Such effects may not be observable in samples more representative of the general population. We note that we did not aim to replicate previous findings pertaining to population subgroups in the current study as our focus was on population level effects.

Impulsivity and intelligence showed unique patterns of association with alcohol use and smoking. Whilst negatively correlated, higher levels of both independently predicted a greater likelihood of alcohol use with a similar magnitude of effect, indicating that each has a distinct impact upon early alcohol use. Regarding smoking, intelligence no longer predicted having smoked a cigarette once impulsivity was included in the model, suggesting that the negative association between intelligence and smoking may be due to shared variance between intelligence and impulsivity–adolescents with higher intelligence may be less likely to smoke because they are less impulsive. Whilst the etiological role of impulsivity-related personality traits in substance use behaviour is well documented [60], few previous studies have tested whether impulsivity predicts alcohol use and smoking after adjusting for intelligence and executive function. Our findings highlight that impulsivity is a robust predictor of early alcohol use and smoking, regardless of individual differences in cognitive ability, albeit with small effect sizes.

Regarding the small to null effects observed overall, it is worth reflecting on how large we might expect the effects of cognitive resource on health behaviour to be in youth, even with highly accurate measurement. Behaviours such as fruit and vegetable consumption and physical activity are influenced largely by caregiver practices during childhood and early adolescence, likely limiting the potential impact of individual differences. Cognitive resource may begin to impact upon these behaviours more strongly once adolescents reach a greater degree of autonomy. Studies with longer follow-up measurement show that higher childhood intelligence results in a lower likelihood of obesity in adulthood, an effect mediated by education and dietary characteristics [29, 61]. Our findings indicate that these patterns may begin to emerge in early adolescence. Future studies may wish to consider whether effects of intelligence on diet and weight in early adolescence persist after accounting for specific caregiver practices, such as making fruit and vegetables available and consuming these themselves [62].

### Strengths and limitations

Key strengths of the current study include the novel contribution of assessing distinct components of executive function and impulsivity alongside intelligence as predictors of multiple health-related behaviours in a large sample, using longitudinal data with sufficient interval between assessment of predictor and outcome variables that reverse causation is unlikely. Intelligence was measured using a scale shown to have high validity and reliability. An additional strength was that our analysis adjusted for socioeconomic characteristics that are associated with the cognitive variables of interest.

A number of weaknesses of the study pertain to the measures used. The alcohol and smoking measures were dichotomous and did not indicate use frequency amongst users. Nevertheless, such measures can be informative. Longitudinal evidence suggests that earlier age of first alcoholic drink increases the risk of heavy alcohol use in young adulthood, and that any experimentation with smoking during adolescence increases the risk of being a smoker 20 years later [63, 64]. Regarding executive function assessment, the measures used typically show weak reliability [65], limiting the magnitude of their observed association with outcome variables. The degree to which the tests given to ALSPAC participants correspond with measures of executive function used with older samples is questionable. For instance, the Stop Signal Task used with older samples typically has a variable rate of stop signal presentation rather than the binary rates used with ALSPAC participants, allowing for greater individual variation in responses. Only one test was used to gauge each executive function, raising the issue of task impurity, whereby task scores reflect aspects of performance other than the executive function of interest [1]. This can be avoided by examining shared variance among multiple measures of each executive function. A further issue relates to whether discrete executive function components are developed enough to be separable in children [2, 3, 21, 66]. However, the modest correlations between executive function measures in this sample did not suggest multicollinearity. Finally, although the sample used was large it was restricted to the Western region of the United Kingdom and findings may not generalise to wider populations.

### Implications

Given the limitations relating to the measurement of executive function, there is a need for replication of our findings with robust assessment of executive function, using multiple tasks to gauge each component. A focus on adult participants, amongst whom executive function is fully developed, is warranted. There is also a need to identify mechanisms by which intelligence, impulsivity, and working memory act upon health-related behaviours and overweight.

From the current findings we are unable to say whether the well documented associations between executive function and health behaviours in adulthood can also be accounted for by intelligence. However, our results raise the possibility that links between specific cognitive capabilities, such as working memory capacity, and certain health behaviours may be explained by general cognitive ability. Researchers investigating how distinct aspects of cognitive resource such as executive function are linked with health behaviour should be aware of overlap with general cognitive ability and personality traits such as impulsivity, and employ a multiple measure approach in future studies.

Our findings have implications for researchers and practitioners developing strategies to improve population health. Given the observed associations between individual differences in cognitive resources and health-related behaviours, interventions that attempt to change these behaviours via cognitive processing reliant on intelligence, impulse control, or working memory may meet with limited success. Alternatively, interventions that can largely bypass this kind of cognitive processing, suggested to include physical environment adaptations [67], may prove more effective. Further research is required to identify whether such interventions do indeed bypass cognitive resources, and to assess their effectiveness. Our results also indicate that interventions that can improve cognitive resource in children may also have beneficial effects on health. Early interventions that provide access to material resources, education, and enriching environments can have a positive impact on children’s cognitive development [68, 69]. The current findings imply that such interventions could also have small indirect effects on improving health behaviour.

In summary, findings from a large UK cohort show that after accounting for intelligence executive function assessed in childhood predicts overweight status but not health-related behaviours in early adolescence, and that impulsivity, a trait related to weak executive function, predicts the onset of alcohol and cigarette use. These results highlight that individual differences in cognitive resource are a potential contributor to health behaviour, yet suggest that the size of effects are small. A better understanding of which aspects of childhood cognitive resource impact upon later health-related behaviours will be beneficial in designing interventions to improve health that do not rely for their effectiveness on cognitive resource.
